# Integration of palliative care in oncology—the intersection of cultures and perspectives of oncology and palliative care

**DOI:** 10.3332/ecancer.2022.1376

**Published:** 2022-04-28

**Authors:** Tonje Lundeby, Marianne Jensen Hjermstad, Nina Aass, Stein Kaasa

**Affiliations:** European Palliative Care Research Centre (PRC), Department of Oncology, Oslo University Hospital, and Institute of Clinical Medicine, University of Oslo, PO Box 4953 Nydalen, 0424 Oslo, Norway

**Keywords:** integration, patient-centred care, palliative care, oncology, culture

## Abstract

Palliative care complements anti-cancer treatment, and may actually improve the therapeutic effect by optimising performance status, functioning, symptom management and quality of life, thus improving compliance to treatment. A series of randomised controlled trials investigating early integration of palliative care and oncology document clear benefits in patient-centred outcomes. Still, palliative care is often introduced late in the disease trajectory, if at all. One reason may be that that palliative care is perceived as end-of-life care only, a too common perception among healthcare providers, patients and the public alike. Another, and maybe the most important, reason is the cultural differences between the two disciplines, oncology and palliative care. While the predominant focus in oncology is treatment and cure of the disease, i.e., a tumour-centred focus, the focus in palliative care is the patient with the disease, i.e., the patient-centred approach. Integration of oncology and palliative care implies that these two cultures approach each other, collaborate and recognise that this is not an either or, but time to collaborate with the best interest of the patients. To accomplish this, an organisational model to provide optimal patient-centred palliative care is necessary at all levels. Such a model must structure the collaborations between different professions, across different levels and the patient flow between the silos in healthcare, and should describe the content of care. Using models like this is not common, and requires changes in systems and cultures on organisational, administrative, educational and individual levels. To successfully achieve profound changes is challenging. For example, it may be hampered by professional autonomy in the different disciplines and hinder collaboration and the achievement of a shared mental model. The use of standardised care pathways may be one way to integrate the tumour-centred and patient-centred approaches, reduce cultural barriers and improve patient care.

*Your patient for the past three years, ‘Julie’, comes for a scheduled consultation. Her metastatic breast cancer has remained stable until now, but is now progressing. She experiences back pain and emotional distress. You feel quite competent in symptom management and know the patient very well; therefore, you are somewhat hesitant to refer her to palliative care; can the relatively inexperienced palliative care physician contribute anything extra? It is anyways difficult to introduce the palliative care services to the patient now – it feels like you are giving up on her and destroying all hope. Besides, you could try a new line of chemo, at least you contemplate it. Therefore, you decide to keep Julie in your care for now, intending to refer her at a later stage of the disease.*

This vignette illustrates what we perceive as a quite common decision-making process in today’s cancer care. It describes a practice in which care is determined by the individual physician’s autonomous choices instead of an evidence-based integrated approach. In this article, we will discuss why this is so and what can be done about it for the best of the patient.

## Introduction

Patient-centred care is the mainstay of palliative care (PC), with the main focus being the patient with the disease and the individual’s perception and preferences related to living with advanced disease. To the best of our knowledge, patient-centred care is marginally implemented into cancer care. This observation contrasts major changes that have taken place in patient care in general and the resources put into anti-cancer treatment.

Despite ample evidence of patient benefits when PC is introduced alongside anti-cancer treatment, many patients, relatives, healthcare providers and the public erroneously misconstrue PC as end-of-life care only [1, 2], which may be one of several causes for unsuccessful implementation. This is prohibitive of patient-centred care if the treatment intention is curative or life-prolonging, and represents a stigma towards PC being a necessary specialty [3].

The most well-documented patient benefits from randomised controlled trials (RCTs) on early provision of PC in the last decades include better symptom management and quality of life, less psychological distress, higher patient and caregiver satisfaction, death in the preferred place, as well as cost-effectiveness and possibly survival benefits [4–11]. Despite small effect sizes, these outcomes are undoubtedly of value for patients and caregivers [12].

Importantly, the design, composition and complexity of the interventions in these RCTs vary, as do the healthcare systems and cultures in which they are expected to work. Still, there are some obvious similarities across studies. First, the focus is on the patient with the disease; the patient-centred approach of PC as opposed to a solitary focus on the cancer; and the tumour-centred approach of mainstream oncology. Furthermore, the RCTs employ different types of patient-reported outcome measures (PROMs) as a part of the diagnostic procedures and to elicit patient perspectives systematically during follow-up. Importantly, high patient scores on symptoms, distress and worries prompt interventions. However, how the PROMs results are implemented into the clinical decision-making processes and individual planning for each patient seems to vary considerably. Furthermore, few studies report substantial changes in routine clinical care after project end.

An important aspect of care planning is related to the design of an individual care plan for the patient, including when and how the different healthcare providers are to be involved. This way of thinking is similar to standardised care pathways, as it delineates the structure, the practical organisation and the content of care delivery, corresponding to the pathway definition of the European Pathway Association [13]. With the organisational aspect of care delivery in mind, a care pathway can define and consequently optimise the responsibilities for the healthcare providers and facilitate communications between different healthcare professionals across care levels within and outside the organisation (e.g., the hospital). This may solve organisational challenges and has been suggested as the method of choice for integration of tumour-centred and patient-centred care [14].

In our opinion, it is noteworthy that despite the favourable study results on integration and clear recommendations and guidelines from ESMO, ASCO and WHO [15–20], few, if any, healthcare systems have implemented this patient-centred structure as part of the clinical routine. Given the complexity of healthcare, there is no single explanation, nor any straightforward solutions to this. In our opinion, it is important to understand and act on the cultural differences between the tumour-centred focus in general oncology and the patient-centred approach of PC as an impediment to integration. Importantly, these should be regarded as complementary parts of quality cancer care, not as a one-or-the-other approach.

## Models of oncology and palliative care

**Figure 1.**
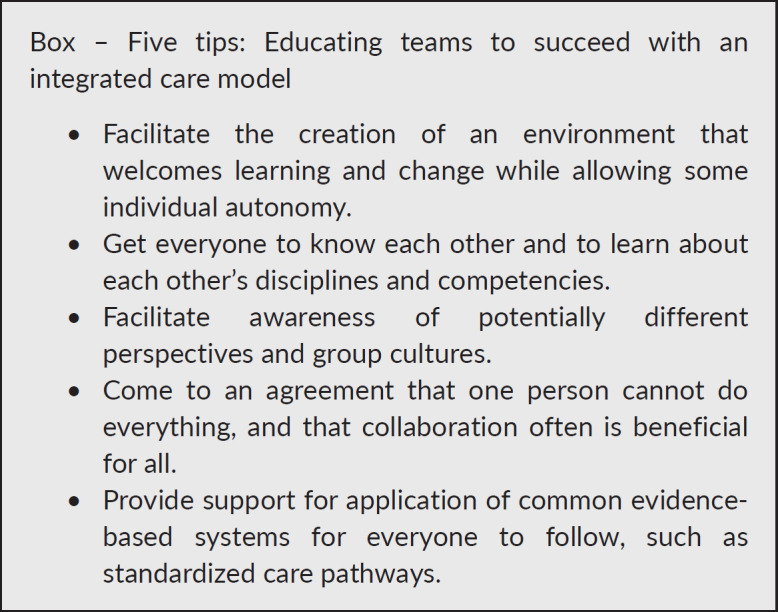
Proposed model of optimal oncology and palliative care provision by different providers across healthcare levels. Source: Kaasa et al [14].

Before discussing the cultural aspects, we refer to the model shown in Figure 1 adapted from the Lancet Oncology Commission ‘Integration of Oncology and Palliative Care’ [14] that shows how PC can be an integrated part of cancer care.

The concept of the model lies in the need to eliminate the gaps between the professional silos in healthcare to provide coherent optimal healthcare at all levels, irrespective of the goal of care being cure, life-prolongation, symptom relief or end-of-life care. Patient-centred PC should be an inherent part of this. As outlined in the figure, SCPs that are based on evidence-based guidelines constitute the core methodology for integration. Noteworthy, this does not mean that the patient is standardised, but that SCPs are the method of choice for the prospective planning and collaboration between primary, secondary and tertiary levels of care, i.e., community care, local hospitals and university hospitals. The underlying premise for succeeding with this model is that PC specialists and oncologists agree on how to overcome the professional boundaries to improve care during the patient’s disease trajectory. They need to reach a mutual agreement on how to collaborate in an integrated oncology and PC pathway that is implemented in routine clinical practice, be it hospital-based or not. This also points to the importance of ascertaining which level of PC is required for the individual patient at different times. But several barriers may hinder such collaboration within and between the levels; and both professional and patient barriers may be culture-dependent.

## Cultures of oncology and palliative care

As mentioned above, oncology and PC represent two disciplines that are not well integrated in most places. Oncology has its origin in mainstream medicine with the objective being to cure illness, i.e., tumour-centred focus. The history of PC, on the contrary, originates from the hospice movement, aiming to improve the quality of life of patients and caregivers at end of life, i.e., the patient-centred approach. As such, the different histories and foci of these two professional disciplines characterise their development over time and their separate cultures and treatment goals. Many factors may explain this. First, the increasing demand for medical treatment and cure in the society, also supported by the medical industry, reinforces the tumour-centred focus at the expense of the patient-centred. This has led to more anti-cancer and intensive therapy at the end of life, often with marginal effects on survival and patient well-being [21, 22]. Unfortunately, many oncologists still consider PC as an end-of-life care issue, and have a perception of self-efficacy to manage patients’ PC needs. As such, many do not recognise the specialised skill set of PC physicians and other PC professionals. Balancing this is the attitude among some PC professionals claiming ‘a monopoly’ of PC excellence. This definitely calls for a cultural behaviour change on both parts.

However, things are not that grim, as we have witnessed a positive development in the collaboration between oncology and PC in the last two decades. First, the patient-centred approach is now largely accepted as a gold standard in healthcare, including oncology and palliative care, albeit not always adequately exercised. Second, palliative care is increasingly becoming a recognised medical field. This is important as it represents an organisational change in delivery of cancer care services with PC as part of the specialist healthcare services. As such, the ground is paved for a closer integration of the two disciplines, but our experience tells us that this is not reality. In fact, PROMs are not routinely used in clinical practice or in clinical studies even when symptom relief is the main goal for the anti-cancer treatment. Still, tumour response is often the primary outcome [23, 24]. Two other examples are the low representation of PC lectures at oncology meetings, frequently arranged as short parallel sessions, and the limited focus on PC in medical school curricula. Paradoxically, the outcomes of many study patients will improve with better symptom management, and as such actually influence the primary study outcomes and patient-centred care as part of a study intervention. Taken together, there is still a way to go, despite a positive development.

## Integration in the intersection: complex systems call for complex solutions

Based on the above, it is obvious that there is not one solution. Changing systems, cultures and clinical routines entails relatively profound changes on organisational, administrative, educational and individual levels, implying a high degree of complexity. Thus, it is essential to focus on the context in which the changes will take place, including the need to define and approach facilitators and barriers on many levels, consider the professional and social contexts and maybe, first, understand the organisational dynamics [25, 26].

While one main pillar to succeed with integration is to achieve a shared mental model [27], it may be hampered by professional autonomy in the different disciplines, obstructing collaboration. In a focus group study [27] conducted a few years ago at our oncology department, the PC physicians stated that the patients were referred too late in the disease trajectory, and that this was dependent upon two things: the culture and tradition of referral of each diagnostic group and, secondly, the individual clinician’s referral routines and preferences. Oncologists, on the contrary, felt competent in providing PC, yet claiming early referrals. If success with integration depends on individual attitudes, renunciation of total and individual autonomy and group culture, we might have to look for solutions addressing these aspects. Importantly, new routines represent a change in the individual mind set and challenge the safe culture one is ‘brought up’ in. It will often automatically cause resistance to implement any change of the system or human behaviour.

This means that change requires consistent top-down and bottom-up involvement and enthusiasm and guarantees that necessary practical and technical aspects work from day 1. Maybe most importantly though is to make sure that the involved professionals realise from within that a change of practice actually represents something better in the way they work, for themselves, the patients and for their colleagues, without too much strain. To understand and accept that change may improve patient outcomes is possible both cognitively and emotionally. But, if the change in practice challenges one’s professional autonomy or culture, increased resistance will occur in many organisations [28].

Going back to you and your patient ‘Julie’ from the introduction the pertinent questions are: Do you and other oncologists or PC physicians consider a closer collaboration across disciplines an opportunity to provide better patient care altogether when in a similar situation? Do you want to collaborate more closely within a common care model? Research shows that close collaboration between professional groups with profound differences in professional identity, may be perceived as destructive to one’s identity [29]. Do referrals to PC collide with the professional autonomy, identity and the oncological cultures? From the palliative care physicians’ view: is there a presupposition of a predominant focus on anti-cancer treatment that hinders collaboration with the oncologist? If so, one way to overcome these barriers may be improved education. Better knowledge about palliative care and oncology might be important for understanding each other’s competence and to succeed with implementation of an integrated care model, shown to be beneficial for the patients.

## Conclusion

Integration of oncology and palliative care implies that two different cultures must approach each other and each other’s mind sets, and that common solutions to the best interest of the patients are reached. But culture is embedded in us, in our behaviour and in the way we think. Thus, creating cultural changes by reconstructing these elements within the relevant groups remains a challenge. Can common practical patient-oriented care plans – the standardised care pathways – be used as a common place ‘to meet’ and plan the care trajectory for each individual patient? We think so.

## List of abbreviations

RCTs – randomised controlled trials

PROMs – patient-reported outcome measures

PC – palliative care

ESMO – European Society for Medical Oncology

ASCO – American Society of Clinical Oncology

WHO – World Health Organisation

## Conflicts of interest

The authors declare no conflicts of interest.

## Funding

None to be declared.
